# Mitotane Concentrations Influence the Risk of Recurrence in Adrenocortical Carcinoma Patients on Adjuvant Treatment

**DOI:** 10.3390/jcm8111850

**Published:** 2019-11-02

**Authors:** Soraya Puglisi, Anna Calabrese, Vittoria Basile, Filippo Ceccato, Carla Scaroni, Chiara Simeoli, Massimo Torlontano, Salvatore Cannavò, Giorgio Arnaldi, Antonio Stigliano, Pasqualino Malandrino, Laura Saba, Barbara Altieri, Silvia Della Casa, Paola Perotti, Paola Berchialla, Giuseppina De Filpo, Letizia Canu, Paola Loli, Giuseppe Reimondo, Massimo Terzolo

**Affiliations:** 1 Internal Medicine, Department of Clinical and Biological Sciences, San Luigi Gonzaga Hospital, University of Turin, 10043 Orbassano, Italy; sorayapuglisi@yahoo.it (S.P.); basile_vittoria@libero.it (V.B.); laura.saba@unito.it (L.S.); oncotrial.sanluigi@gmail.com (P.P.); giuseppe.reimondo@unito.it (G.R.); massimo.terzolo@unito.it (M.T.); 2 Endocrinology Unit, Department of Medicine DIMED, University-Hospital of Padua, 35128 Padova, Italy; ceccato.filippo@gmail.com (F.C.); carla.scaroni@unipd.it (C.S.); 3 Endocrinology Unit, Department of Clinical Medicine and Surgery, University of Naples Federico II, 80131 Naples, Italy; simeolichiara@gmail.com; 4 Endocrinology Unit, Hospital “Casa Sollievo della Sofferenza”, IRCCS, 71013 San Giovanni Rotondo, Italy; m.torlontano@operapadrepio.it; 5 Department of Human Pathology of Adulthood and Childhood ‘G. Barresi’, University of Messina, 98125 Messina, Italy; cannavos@unime.it; 6 Division of Endocrinology, Department of Clinical and Molecular Sciences (DISCLIMO), Polytechnic University of Marche, 60121 Ancona, Italy; gioarnaldi@gmail.com; 7 Endocrinology, Department of Clinical and Molecular Medicine, Sant’Andrea Hospital, Sapienza University of Rome, 00189 Roma, Italy; antonio.stigliano@uniroma1.it; 8 Endocrinology Division, Department of Clinical and Experimental Medicine, ARNAS Garibaldi, University of Catania, 95122 Catania, Italy; linomalandrino@gmail.com; 9 Division of Endocrinology and Metabolic Diseases, University-Hospital Gemelli, IRCSS, Catholic University of the Sacred Heart, 00168 Rome, Italy; altieri.barbara@gmail.com (B.A.); silvia.dellacasa@unicatt.it (S.D.C.); 10 Statistical Unit, Department of Clinical and Biological Sciences, University of Turin, 10143 Orbassano, Italy; paola.berchialla@unito.it; 11 Endocrinology Unit, Department of Experimental and Clinical Biomedical Sciences, University of Florence, 50134 Florence, Italy; giuseppina.defilpo@unifi.it (G.D.F.); letizia.canu@unifi.it (L.C.); 12 Endocrinology, Hospital Niguarda Ca’ Granda, 20121 Milan, Italy; paola.loli@ospedaleniguarda.it

**Keywords:** adrenocortical carcinoma, mitotane, prognosis, recurrence, survival

## Abstract

Mitotane is used as a post-operative adjuvant treatment for patients with adrenocortical carcinoma. Monitoring of plasma mitotane concentrations is recommended, but we do not know what impact target concentrations have on patient outcome. To answer this question, we retrospectively analyzed patient records in the Lysosafe Online^®^ database (HRA Pharma, France) for patients who were treated for ≥6 months and who had ≥3 measurements of plasma mitotane levels during follow-ups at 11 tertiary centers in Italy from 2005 to 2017. We identified 110 patients treated with adjuvant mitotane for a median of 46 months (IQR, interquartile range, 28–62) with a median maintenance dose of 2.0 g/day (IQR 1.5–2.5). Achievement of target mitotane concentrations (≥14 mg/L) required a median of 8 months (IQR 5–19). Female sex was associated inversely with the dose, while body mass index (BMI) was correlated positively. Multivariate analysis showed that the Ki67 index and time to achieve the target range of plasma mitotane were independent predictors of recurrence-free survival (RFS). In a separate multivariate model, considering only the maintenance phase (month 7 to month 36, M7–M36) of treatment, the time in the target range of plasma mitotane was associated with a significantly lower risk of recurrence (Hazard Ratio, HR = 0.93; 0.88–0.98, *p* < 0.01). The prognostic implications of the time in target range and the time needed to reach target mitotane concentrations support the use of mitotane monitoring and may inform practice.

## 1. Introduction

Treatment of adrenocortical carcinoma (ACC) is based on surgery that is usually the first and most effective therapeutic strategy [1,2,3,4]. Complete removal of the tumor may lead to a cure; however, ACC has a strong propensity to recur after surgery, even when R0 (free resected margins) operations are completed by skilled surgeons [5]. Therefore, the European Society of Endocrinology (ESE) - European Network for the Study of Adrenal Tumors (ENSAT) guidelines on management of ACC indicate adjuvant mitotane treatment for patients at high risk of recurrence, although they recognize a low level of evidence, based only on retrospective, non-randomized studies [6]. It is recommended to regularly monitor plasma mitotane levels during treatment with the aim of maintaining levels >14 mg/L [6], based on studies suggesting a link between high mitotane levels and drug efficacy [7,8,9]. However, the evidence supporting a target range when mitotane is used as an adjuvant measure is limited and conflicting [8,10], and this may be due to the challenge in assessing the optimal exposure to mitotane in chronic treatments. 

To evaluate whether plasma mitotane concentrations have prognostic implication in ACC patients on adjuvant mitotane treatment, we reviewed retrospectively the experience of 11 tertiary centers for the care of ACC patients in Italy, adopting a novel method to assess target mitotane concentrations. 

## 2. Experimental Section

For this study, we invited 13 tertiary centers for the care of ACC patients in Italy. Eleven centers accepted the invitation to participate in the survey, providing clinical, pathological, and biochemical data of all ACC patients who had been proactively followed at the center and treated with adjuvant mitotane. We retrieved data of patients who were treated from July 2005 to July 2015. Follow up for this study was closed in December 2017. The institutional ethics committee of all centers approved the study, and all patients signed written informed consent (for patients under 18 years-of-age, parental written consent was provided). 

Inclusion criteria of the study were as follows: age ≥16 years, pathologically confirmed diagnosis of ACC, complete macroscopic resection, availability of pre-operative and post-operative computed tomography (CT) or magnetic resonance imaging (MRI) scans, complete follow-up information, treatment with mitotane (all patients received the same mitotane formulation, Lysodren^®^ 500 mg tablets) for ≥6 months and with ≥3 measurements of plasma mitotane concentrations reported on the Lysosafe Online^®^ database. Exclusion criteria were as follows: incomplete tumor staging, history of other previous or concomitant malignancies, R2 (macroscopic invasion of resected margins) resection, incomplete follow-up information, concomitant adjuvant chemotherapy and radiotherapy or both, and concomitant treatment with any drug specifically directed against ACC.

Patients’ charts were reviewed and the following information was retrieved for the study: gender, age, body mass index (BMI), date of diagnosis, hormone secretion, ACC stage, pathology report, date of recurrence, last follow-up or death. Date of diagnosis was defined as the date of surgery. Biochemical confirmation of hormone excess was requested to categorize an ACC as hormone secreting. Tumor stage was established according to the ENSAT classification (I and II, confined tumor; III, positive lymph nodes or infiltrating neighboring organs/veins without distant metastases; IV, distant metastases [11].

Date of recurrence was defined as the date of radiological evidence of a new lesion. A questionnaire was sent to the participating centers to retrieve the information requested for the study; moreover, centers were asked about indications, timing of initiation and discontinuation, reasons for discontinuation and dose regimen of adjuvant mitotane treatment, and follow-up modality. The schedule of follow-up visits was similar between centers, with an early assessment between 3–6 weeks after treatment initiation including physical exam, plasma mitotane monitoring, and biochemical work-up. Evaluations were afterwards scheduled every 3–4 months with physical exam, full body imaging, plasma mitotane monitoring, and biochemical work-up for at least 3 years. After this time limit, timing of the follow-up visits was individualized according to the preferences of the patients and physicians. Duration of treatment was calculated from the date of initiation of mitotane therapy until ACC recurrence, or discontinuation of treatment, or the end of follow-up, whichever occurred first.

For the analysis of plasma mitotane concentrations, we separately considered the first six months of therapy (M0–M6) because this is the period when mitotane dose is progressively increased to attain target levels and, as a consequence, mitotane concentrations are highly variable. During the first six months, we analyzed the correlation between mitotane concentrations and the month of therapy and drug dose. We considered the period from month 7 to month 36 (M7–M36) as the maintenance phase, because mitotane dose is usually stable in chronic treatment. We set the time point at the 36th month, since the timing of follow-up visits was more consistent between centers during this period. In a multivariate regression analysis, we assessed the correlation between plasma mitotane concentrations recorded during M7–M36 and patient sex, age, and BMI. We also calculated the time in target range (TTR) during M7–M36, defined as the number of months in which mitotane concentrations were ≥14 mg/L, a value considered as the lower limit of the target range [7,8,9], for all patients. Based on the concept of the “time in therapeutic range” used for monitoring warfarin therapy [12], we assumed that a linear relationship existed between consecutive values when a measurement was not available.

Mitotane concentrations were retrieved from the Lysosafe Online^®^ database, available at www.lysosafe.com. Lysosafe Online^®^ is a login-protected website that stores mitotane plasma concentrations of patients treated by physicians who have registered with the Lysosafe^®^ service, a free-of-charge service of measurement of plasma mitotane concentrations in ACC patients offered by HRA Pharma to European prescribers since 2005 and associated with the use of Lysodren^®^. Samples are collected at the centers, sent to a centralized laboratory, extracted by precipitation with ethanol, and tested by a standardized gas chromatography/mass spectrometry method. Plasma mitotane values of any patient are available for the treating physician on www.lysosafe.com, in a historical and graphic plot that matches mitotane levels with the relative Lysodren^®^ dose. Patient data are anonymous during the whole process since patients are recorded using an acronym and their date of birth.

All communication concerning the study between centers was by email, and a meeting was organized to make the process of data capture more homogeneous.

Categorical data are presented as counts and percentages. Continuous data are presented as medians and interquartile ranges (IQR). Differences in categorical variables were analyzed by means of the chi-squared test or Fisher test as appropriate, while differences in continuous variables were analyzed by the Mann–Whitney U test. Correlation analyses were determined by calculating the Spearman’s R coefficient. Multiple regression analysis was done as appropriate. The survival curves were estimated with the Kaplan–Meyer product limit method. Recurrence-free survival (RFS) was calculated from the time of initial surgery to the first radiological evidence of recurrence. Overall survival (OS) was calculated from the date of initial surgery to the date of death. Patients who did not experience either of those events (recurrence or death) were censored at the date of the last follow-up visit for the specific survival analysis. Cox proportional hazards regression models were fitted to determine prognostic factors on RFS and OS. The following potential predictive factors for either RFS or OS were investigated: patient sex and age, tumor stage, hormone secretion, Weiss score, Ki67 index, and the time elapsed to get the first plasma mitotane level at target. Stratification of patients into risk groups was achieved through the maximally-selected log-rank statistics approach, which provides the value of a cut-off point corresponding to the most significant relation with outcome [13]. We did a second multivariate analysis to assess whether the TTR of mitotane concentrations between M7 and M36 was an independent factor influencing RFS or OS. All reported *p* values are two-sided. The *p* values less than 0.05 were considered as statistically significant. The statistical analyses were performed with Statistica (StatSoft) (Dell Software, Round Rock, Texas, USA) and R version 3.5.1 (R Core Team, USA).

## 3. Results

**Figure 1:**
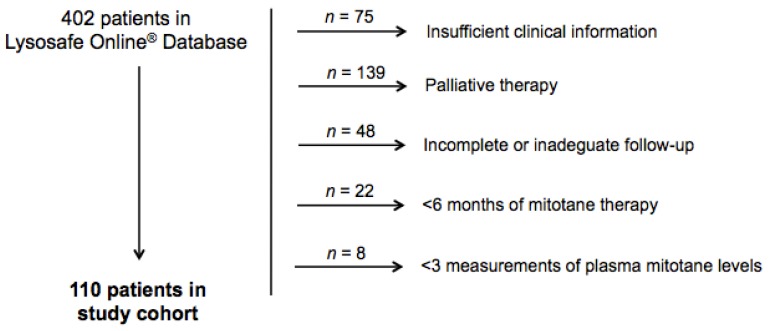
Study cohort.

From a total of 402 ACC patients on the Lysosafe^®^ Online database, 110 patients fulfilled inclusion/exclusion criteria and were retrospectively included in the study (Figure 1). Baseline characteristics of patients are reported in Table 1.

The median follow-up was 63 (39–94) months. Adjuvant mitotane treatment was initiated after a median time of 1 (1–2) month from the first surgery in 102 patients (92.7%) and after surgical treatment of a recurrence in eight patients (7.3%). Median duration of treatment was 46 (28–62) months. The adjuvant therapy was discontinued permanently in 59 cases (53.6%), of which 36 (61.0%) were for end of treatment after a median time of 58 (45–62) months. Other causes of treatment discontinuation were toxicity (*n* = 5), patient’s decision (*n* = 4), concomitant diseases (*n* = 3), or other/not available data (*n* = 11). Characteristics of mitotane treatment at the time of permanent discontinuation for toxicity are reported in Table 2. Gastrointestinal symptoms included nausea and diarrhea, whereas neurological manifestations were dizziness and confusion.

All centers reported recommending adjuvant mitotane treatment in all ACC patients following operation, with the exception of three centers, where treatment was offered to high-risk patients only. All centers but one reported using a low-dose regimen with minimal variations in the starting dose (1–2 g/day), while the velocity of further dose increments varied among centers. Maintenance dose was guided by results of mitotane monitoring and patient tolerability. 

During the period M0–M7, plasma mitotane levels increased progressively, being significantly correlated with month of therapy (r = 0.6, *p* < 0.0001) and mitotane doses (r = 0.13, *p* < 0.034). Achievement of target mitotane levels required a median time of 8 (5–19) months from start of therapy, while 11 patients (10%) never achieved levels ≥14 mg/L. 

The median mitotane dose in the maintenance phase was 2.0 (1.5–2.5) g/day. In a multiple regression analysis, sex (*β* = -0.23, *p* = 0.02) and BMI (*β* = 0.22, *p* = 0.02) were correlated with median doses of mitotane, implying that female sex was associated inversely with the dose, while BMI was correlated positively. In the group of 102 patients who started adjuvant mitotane therapy after the first surgery, recurrence occurred in 39 (38.2%) of cases. Median RFS was not reached, and the median follow-up time for RFS was 54 months (27–78). Multivariate analysis showed that the Ki67 index and time to the first mitotane level at target were independent predictors of RFS (Table 3). 

**Figure 2:**
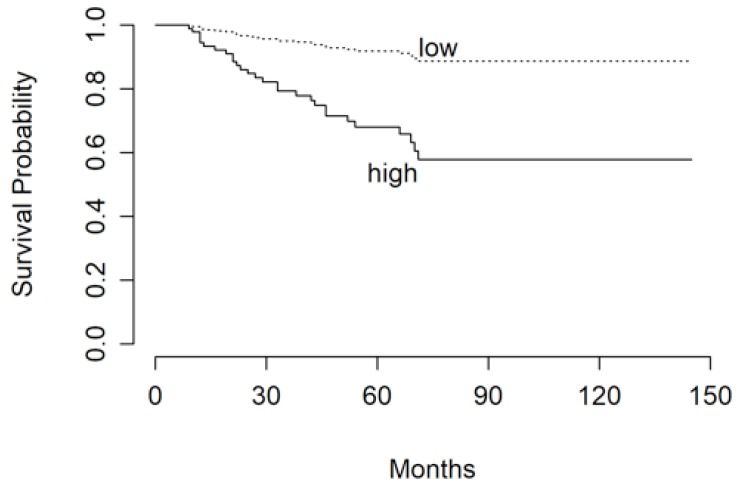
RFS of patients stratified in “low” and “high” risk groups according to Ki67 indices of ≤10% and >10%, respectively.

**Figure 3:**
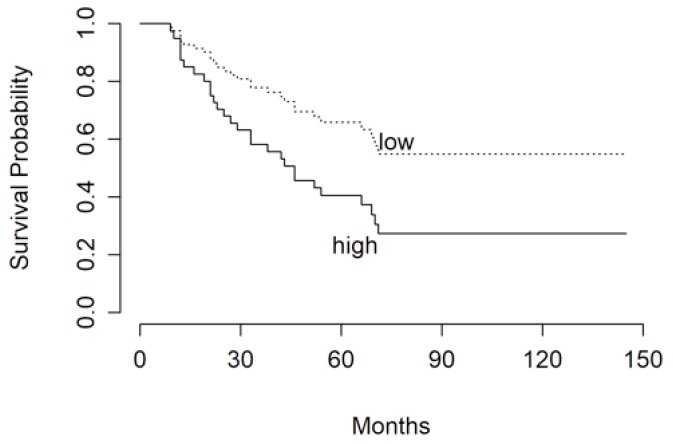
RFS of patients stratified in “low” and “high” risk groups according to the time needed to reach target mitotane concentrations of ≤17 and >17 months, respectively.

We identified a cut-off value for the Ki67 index at 10% and for time to reach target mitotane concentrations at 17 months, which were able to differentiate significantly patients for their risk of recurrence (Figure 2 and Figure 3).

In a separate multivariate model considering only the maintenance phase (M7–M36) of treatment, TTR was associated with a significantly lower risk of recurrence (Hazard Ratio, HR = 0.93; 95% CI, 0.88–0.98; *p* < 0.01). Death occurred in 22 cases (20%). Median OS was not reached, and the median follow-up time for OS was 70 months (49–100). We did not find any predictor of the risk of death due to the low number of events.

## 4. Discussion

The present study confirms that a remarkable number of patients with ACC who are operated on with radical intent are destined to relapse [2,5,14,15,16,17,18]. ACC recurrence portends a worse prognosis and has a huge impact on quality of life; therefore, investigators have considered the use of adjuvant mitotane therapy following surgical operation. However, a limited number of often small-sized studies reporting the outcome of adjuvant mitotane is available [19,20,21,22]. 

In the present study, we explored the relationship between target mitotane concentrations and patient outcome using the TTR, analogous to warfarin treatment [12]. Some previous studies used the peak mitotane level, which cannot give an adequate representation since it is a measurement at a single point in time [7]. In other studies, the percentage of mitotane measurements in a range was used, but this method has the caveat of being strongly dependent on the number of available measurements. This may introduce a bias when comparing patients with different durations of follow-ups, which may be quite prolonged in the case of adjuvant therapy. Moreover, there is no evidence to define what percentage level identifies a good exposure to mitotane. These methodological issues may have contributed to the discrepancy in the literature concerning adjuvant treatment [8,10]. In the present study, we calculated the TTR, which in our opinion gives a more adequate representation of chronic exposure to mitotane, and analyzed the results in a multivariate analysis without predefining arbitrary cutoff values. We found an inverse relationship between the TTR and risk of ACC recurrence, implying that the greater the TTR, the lower the risk. This finding supports the clinical value of mitotane monitoring and the concept of target doses in the adjuvant setting [6]. Although it is plausible that lower mitotane concentrations may be effective in an adjuvant treatment, we analyzed only the level of 14 mg/L, because this was the target in our practice. 

The time needed to achieve target mitotane concentrations also has an impact on the risk of ACC recurrence: a longer time was associated with higher risk. This is consistent with the concept that mitotane is a slow-acting drug in relation to the achievement of significant plasma levels. Due to the very cautious dose titration in the starting phase of treatment employed in many centers, the time needed to get into the target range was exceedingly long. We identified a time point at 17 months to achieve target concentrations, which significantly differentiates patients for their risk of recurrence. The present findings call for a change in practice, aiming for a faster rise in mitotane levels and strengthening the value of mitotane monitoring. However, the potential danger of a rapid increment in mitotane dosing, which may result in important toxicity with consequent loss of compliance to treatment, should be considered [20].

We found only a weak relationship between mitotane dose and its plasma concentrations during the first phase of treatment, and this finding is in agreement with the concept that individual differences in mitotane metabolism and other still unknown factors influence plasma concentrations [23,24]. Interestingly, higher doses were employed in men and in patients with greater BMI and these novel findings matter for clinical practice. 

In our cohort, toxicity associated with adjuvant mitotane was acceptable, though we acknowledge the fact that due to the study inclusion criteria we did not capture the patients who eventually discontinued mitotane in the first six months. Severe toxicity leading to permanent treatment discontinuation was recorded in only five patients on chronic therapy, and this is likely due to the low doses (median dose of 2 g/day) used to continue treatment in the maintenance phase. Thus, the present study shows that a few patients cannot tolerate adjuvant mitotane following the first months of treatment, proving than a careful follow-up is necessary. Despite mitotane having a reputation for being a challenging drug to manage [17], adjuvant mitotane treatment is feasible when patients are managed in expert centers. However, some patients were unable to tolerate the drug, exhibiting neurological toxicity even when exposed to “normal” mitotane concentrations, confirming the relevance of individual factors in mitotane metabolism. 

Strengths of the present study are the thorough characterization of adjuvant mitotane treatment of ACC patients following surgical removal of the tumor and the large data set, considering the rarity of the disease. This allowed for the capture of details of mitotane treatment that were not available in previous studies and led to observations that may be useful to informing future practice. However, we should acknowledge the limits of a retrospective analysis, and that our results are not generalizable to patients who discontinue treatment within six months for intolerability or patients with early ACC recurrence. The inclusion criteria of the study produced an immortal time of six months that may have enriched our series of a higher number of low-risk ACC compared to recently published series [10,25].

## 5. Conclusions 

In conclusion, a low-dose mitotane regimen is generally well tolerated but has the drawback of needing quite a long time to reach the target concentrations. The observation that the TTR of mitotane and the time to the first level in range are associated to the risk of recurrence is novel and has practical importance. Thus, the use of mitotane monitoring and the value of target mitotane concentrations are justified by this analysis of real practice.

## Figures and Tables

**Table 1:** Baseline features of patients. IQR = interquartile range. BMI = Body Mass Index.

Characteristics	Valid Cases (*N*)	Values
**Gender**, *N* (%)	110	
Male		43 (39.1%)
Female		67 (60.9%)
**Age at diagnosis**, year	110	
Median (IQR)		47 (35–58)
**BMI**, kg/m^2^	95	
Median (IQR)		24.7 (21.9–29.4)
**Tumor stage**, *N* (%)	110	
Stage I		11 (10%)
Stage II		80 (72.7%)
Stage III		17 (15.5%)
Stage IV		2 (1.8%)
**Hormone secretion**, *N* (%)	106	
Yes		58 (54.7%)
No		48 (45.3%)
**Weiss score**	90	
Median (IQR)		6 (5–7)
**Ki67**	95	
Median (IQR)		17 (6.5–30)
≤10%		32 (33.7%)
>10%		63 (66.3%)

**Table 2:** Characteristics of mitotane treatment at the time of permanent discontinuation for toxicity. Legend: GI = gastrointestinal, NEU = neurological.

Patients	Mitotane Levels (mg/L)	Mitotane Dose (g/Day)	Duration of Treatment (Months)	Type of Toxicity
**1**	12.7	1.5	23	GI
**2**	13.2	2.0	45	GI
**3**	9.2	1.5	31	NEU
**4**	10.6	2.5	19	NEU
**5**	12.4	2.5	18	GI/NEU
Median (IQR)	12.4 (10.6–12.7)	2.0 (1.5–2.5)	23 (19–31)	

**Table 3:** Univariate and multivariate analysis of predictive factors for recurrence-free survival (RFS). HR = Hazard Ratio. The bold indicates the statistically significant values.

Title	Univariate Analysis			Multivariate Analysis		
Factor	HR	95% CI	*p* Value	HR	95% CI	*p* Value
Gender ^1^	1.35	0.71–2.54	0.358	-	-	-
Age at diagnosis	1.10	0.67–1.80	0.709	-	-	-
Tumor stage ^2^	2.17	1.03–4.59	**0.042**	-	-	-
Hormone secretion ^3^	0.79	0.42–1.52	0.486	-	-	-
Weiss score	1.60	1.03–2.48	**0.038**	-	-	-
Ki67 index	1.49	1.06–2.10	**0.023**	4.49	1.57–12.84	**0.005**
Time to first level at target	1.22	1.00–1.50	0.053	1.48	1.06–2.07	**0.020**

Reference categories: ^1^ Male gender, ^2^ Stage III–IV, ^3^ Non-secreting tumors.
